# Optimizing diets for finishing pigs: balancing ideal protein to enhance growth performance and reduce environmental impacts

**DOI:** 10.1093/tas/txaf141

**Published:** 2025-10-23

**Authors:** Christina C Mulvenna, Ursula M McCormack, Kelvin J McCracken, Sam Smyth, Fred J Gordon, Violet E Beattie, Raymond Bradford, Marvelous Sungirai, Elizabeth Magowan, Ramon Muns, Elizabeth Ball

**Affiliations:** Sustainable Agri-Food Sciences Division, Agri-Food and Biosciences Institute, Hillsborough BT26 6DR, United Kingdom; Sustainable Agri-Food Sciences Division, Agri-Food and Biosciences Institute, Hillsborough BT26 6DR, United Kingdom; Devenish Nutrition, Belfast BT3 9AR, United Kingdom; John Thompson & Sons, Limited, Belfast BT15 3GW, United Kingdom; John Thompson & Sons, Limited, Belfast BT15 3GW, United Kingdom; Devenish Nutrition, Belfast BT3 9AR, United Kingdom; Preferred Capital Management, Lisburn BT27 4EL, United Kingdom; Sustainable Agri-Food Sciences Division, Agri-Food and Biosciences Institute, Hillsborough BT26 6DR, United Kingdom; Sustainable Agri-Food Sciences Division, Agri-Food and Biosciences Institute, Hillsborough BT26 6DR, United Kingdom; Sustainable Agri-Food Sciences Division, Agri-Food and Biosciences Institute, Hillsborough BT26 6DR, United Kingdom; Sustainable Agri-Food Sciences Division, Agri-Food and Biosciences Institute, Hillsborough BT26 6DR, United Kingdom

**Keywords:** amino acids, crude protein, finisher pigs, growth performance, nitrogen excretion

## Abstract

To investigate the performance of pigs offered diets with reduced crude protein (CP), but supplying ideal protein with different limiting amino acids (AAs), 280 pigs (PIC 337 × (Landrace × Large White)) were randomly allocated to 1 of 4 treatments (with 7 pen replicates per treatment) balanced for weight and sex. The pigs were penned in mixed-sex (5 females and 5 males) pen groups of 10 and offered treatment diets from 12 wk of age (≈40 kg liveweight) to slaughter (≈112 kg liveweight). The control diet had 175 g/kg CP formulated to contain ideal protein to lysine (Lys), methionine (Met), and threonine (Thr) and the remaining 3 diets contained 150 g/kg CP. Ideal diet 1 had ideal protein to Lys, Met, Thr, tryptophan (Trp), valine (Val), isoleucine (Ile) and arginine (Arg). Ideal diet 2 had ideal protein to Lys, Met, Thr and Trp, and Ideal diet 3 had ideal protein to Lys, Met and Thr. All diets had 13.85 MJ/kg of digestible energy. Feed intake, average daily gain (ADG), and feed conversion ratio (FCR) were measured. ADG was higher (*P* = 0.021) between 12 and 15 wk of age in pigs fed Ideal 1 (786 g/d) and 2 (755 g/d) diets than pigs fed the control diet (682 g/d), resulting in a heavier body weight (≈2 kg) at 15 wk. The FCR was improved (*P* = 0.033) between 12 and 15 wk of age in pigs fed Ideal 1 diet (2.08) than that of the control diet (2.41). Diet had no effect (*P* > 0.05) on overall pig performance between 40 and 112 kg liveweight. Cold carcass weight was heavier (*P* = 0.006) in pigs fed Ideal diet 1 than pigs fed other diets. A dietary reduction in CP by 25 g/kg fed to boars and gilts from 40 kg reduced N intake (*P* = 0.003) and excretion (*P* < 0.001) by 15% and 24%, respectively, with percentage nitrogen retention (average = 41.9%) remaining unaffected across treatments (*P* = 0.891). Formulating feed down to Ile and Val had production benefits (higher ADG and better FCR) in the early finishing period, although no effect was observed during the late finishing stages. Economically, Ideal 3 diet with 150 g/kg CP balanced for ideal protein to Lys, Met, and Thr was the most cost-effective. We suggest that, while formulating diets down to 6 AAs is not essential, there is need to pay attention to early finishing diets to yield production benefits. We provide further evidence in support of reducing dietary CP without sacrificing overall pig production performance, at the same time, lowering the negative environmental impact.

## Teaser

In this study, we report sustainable pig production methods through diets with reduced crude protein content that maintain growth performance and decrease nitrogen excretion to achieve environmental targets. This research represents a major development toward sustainable and economical methods for raising pigs.

## Introduction

Despite pig production having a relatively low environmental impact in comparison with ruminant production, the increasing demand for pig meat and the intensive system of production means there needs to be a focus on lowering environmental pollutants. Of particular importance is the excretion of excess nitrogen (N) (and subsequent ammonia), which can lead to acidification and eutrophication (De Vries and De Boer 2010). There are several methods by which N excretion can be reduced; improved feed efficiency and growth performance through advances in genetics and management, improved N utilization and through reduced N intake by lowering dietary crude protein (CP) (Webb et al. 2014; Ottosen et al. 2021). Reducing CP content of pig diets while maintaining production performance has both economic and environmental benefits (Wang et al. 2018). For pigs, the need for CP is basically the need for amino acids (AAs), which are the building blocks of proteins and are essential for normal bodily function throughout the lifetime of the pig (Van Milgen and Dourmad 2015). Therefore, a reduction in dietary CP content requires the supplementation of essential AA to maintain optimum levels of production in pigs. The ratio of AA inclusion is critical and if CP is too low, then there is an undersupply of nonessential AA required for protein deposition (Lynegaard et al. 2021). Lysine (Lys) is generally the first limiting AA in pig diets and has been set as the reference AA, with all other AAs expressed as a percentage relative to it (Liao et al. 2015). The “Ideal protein concept” assumes that the ideal dietary profile contains the optimum balance of all AA necessary for maintenance and efficient production at different growth stages (Van Milgen and Dourmad 2015). Therefore, careful formulation of diets to meet the ideal protein (IP) requirement can ensure pig performance and health and minimize the cost and environmental excretion of nitrogen. However, in practice, all essential AA are not synthetically available, or their inclusion is not economically viable and even some nonessential AA may be undersupplied if levels of dietary CP are too low. For example, while arginine (Arg) can be synthesized *de novo* there is evidence to suggest that if the building blocks for Arg are undersupplied (ie insufficient CP) then Arg can become a limiting AA for growth (Wu et al. 2014).

The order of limiting AA in diets for pigs depends on the stage of production of the pig. Typically for growing/finishing pigs offered cereal/soyabean meal-based diets the order of limiting AA is Lys, Methionine (Met), Threonine (Thr), Tryptophan (Trp), Valine (Val), Isoleucine (Ile) and Leucine (Leu), Phenylalanine (Phe), and Histidine (His) (Van Milgen and Dourmad 2015). However, others have reported that the first 6 limiting AA are Lys, Met, Thr, Trp, Ile, and Val with some debate as to the order of Ile and Val (Whittemore et al. 2003; Wang et al. 2018). For growing/finishing pigs, the majority of the research investigating IP, AA supply and lowering CP has focused on Lys levels and balancing IP down to Thr in cereal/soyabean meal-based diets. Diet production costs and AA availability have limited the balancing of all essential AA in the IP ratio in commercial practice, although the theory of complete IP balance is an accepted principle. With increased availability of more synthetic AAs and the environmental benefits of reducing dietary CP further, there is a need to identify the optimum level of IP balance in terms of specific AA required for optimum performance.

Much of the research conducted on lowering CP has been on gilt/castrate systems with lower CP requirements *per se* than that needed by gilt/entire boar systems. Nonetheless, the principle has been proven that dietary CP can be reduced to a certain level when supplemented with essential AA without detrimental effects on performance. Wang et al. (2018) stated that in general, CP can be reduced by up to 3% below quoted standards (National Research Council 2012) if diets are supplemented with the first 4 limiting AA; Lys, Met, Thr, and Trp. It is suggested that supplementation beyond these 4 AA can allow for further reductions in dietary CP but there is a lack of information on balancing IP beyond Trp in diets for finishing gilts and boars with a high genetic rate of protein deposition. Furthermore, there is controversy on the effect of balancing to Val and Ile with Roux et al. (2011) reporting that Val and Ile did not restore performance to levels equivalent to the positive high protein control and Powell et al. (2011), Zheng et al. (2016) and Liu et al. (2017) reporting benefits to balancing lower CP diets down to Val and Ile. From previous work, it is clear that to ensure optimum pig performance of finishing pigs in a production system of gilts and entire boars using reduced dietary CP levels, the optimum supplementation of AAs to meet “Ideal protein balance” needs to be achieved, while also considering the inevitable increased diet costs. To achieve this, the overall aim of this work was to compare the growth performance, feed efficiency and fecal consistency of finisher pigs offered diets reduced in CP content differing in levels of supplementary AA and formulated down to the first 6 limiting AA.

## Materials and methods

### Statement of institutional animal care and use committee

Animal procedures were approved by the Animal Welfare and Ethical Review Body at Agri-Food and Biosciences Institute (AFBI) Hillsborough. The experiment was conducted at AFBI in compliance with the UK Animals Scientific Procedures Act, 1986 (Hollands 1986).

### Experimental design and diet composition

A total of 280 pigs (PIC 337 × (Large White × Landrace)) were used in the study, 20 boars and 20 gilts per batch, over a total of 7 batches. At 10 wk of age, pigs were randomly assigned to 1 of 4 treatment diets; (i) Control (175 g/kg CP formulated to meet the amino acid requirements of pigs based on the National Research Council (National Research Council 2012) recommendation, with IP balanced to Lys, Met, and Thr), (ii) Ideal 1 (150 g/kg CP, with IP balanced to Lys, Met, Thr, Trp, Val, and Ile), (iii) Ideal 2 (150 g/kg CP, with IP balanced to Lys, Met, Thr, and Trp), and (iv) Ideal 3 (150 g/kg CP, with IP balanced to Lys, Met, and Thr). All diets were formulated to contain 13.85 MJ/d of digestible energy, which is slightly above recommendations of the British Society of Animal Science (National Research Council 2012). The ingredient composition and feed formulation are shown in Tables 1 and 2. The diets formulated in this study by fluctuating ingredients reflected cost-saving feeding strategies that are more accessible to commercial pig producers at the expense of synthetic amino acids (AAs), which could increase feed costs. Moreover, this strategy mimicked practical feeding conditions for commercial pig producers (Van Milgen and Dourmad 2015). DL-methionine and L-threonine synthetic AAs were included to meet amino acid requirements, whereas cysteine and glutamate were adjusted with natural ingredients (soybean meal, rapeseed meal, and maize). Pigs were housed in pens of 10 (5 boars and 5 gilts/pen) giving 7 pen replicates per treatment. Single-phase feeding was adopted in this experiment whereby pigs were offered the same diets from 12 wk of age (≈40 kg liveweight) until slaughter (≈112 kg liveweight) at 22 wk in order to compare the effects of reduced CP and lower AA balancing on growth performance, N excretion and economic outcomes. Previous to 12 wk of age, all pigs were offered the same commercial dietary regime with diets formulated to recommendations (National Research Council 2012). Each pen (3.25 × 2.00 m; fully slatted concrete) was equipped with single space wet and dry feeders and a nipple drinker, providing all pigs with ad libitum access to feed and water. Pig live weights and feed usage were recorded every 3 wk. Ventilation was provided by automatically controlled natural ventilation, and the internal temperature was maintained on average at 18 °C. Lighting was by natural daylight and artificial lighting during the day, and a low level of artificial lighting during the night (18 h L:6 h D).

**Table 1.** Ingredient composition (%) of experimental diets.

Ingredient (%)	**Control** ^a^	**Ideal 1** ^b^	**Ideal 2** ^c^	Ideal 3^d^
**Barley**	30.0	30.0	21.1	21.5
**Wheat**	10.0	17.3	24.6	24.1
**Maize**	27.0	30.0	30.0	30.0
**Pollard**	5.0	4.4	5.0	5.0
**Rapeseed**	5.0	0.0	5.0	5.0
**Soyabean meal**	19.3	13.7	10.1	10.2
**Vegetable oil (mix)**	0.5	0.5	0.5	0.5
**Vegetable oil (spray)**	0.5	0.5	0.5	0.5
**Limestone**	1.30	1.23	1.24	1.24
**Mono di-calcium phosphate**	0.35	0.58	0.44	0.44
**Salt**	0.63	0.44	0.43	0.43
**L-Lysine HCl**	0.24	0.52	0.53	0.53
**DL-Methionine**	0.06	0.14	0.11	0.11
**L-Threonine**	0.00	0.18	0.17	0.17
**L-Tryptophan**	0.00	0.03	0.03	0.00
**L-valine**	0.00	0.10	0.00	0.00
**L-Isoleucine**	0.00	0.05	0.00	0.00
**Minerals and vitamins^e^**	0.30	0.30	0.30	0.30

a Control: 175 g/kg crude protein (CP).

b Ideal 1: 150 g/kg CP with IP balanced to Lys, Met, Thr, Trp, Val, Ile and Arg.

c Ideal 2: 150 g/kg CP with IP balanced to Lys, Met, Thr and Trp.

d Ideal 3: 150 g/kg CP with IP balanced to Lys, Met, and Thr.

e Vit A 8000 iu, Vit D_3_ 2000 iu, Vit E 75 g, Vit K 1 g, Vit B_2_ 2 g, Vit B_12_ 20 mg, Biotin 0.05 g, Cal-D-Pan 8 g, Nico 10 g, iodine 1 g, Selenium 0.15 g, Iron 100 g, Manganous 30 g, Copper 12.5 g, Zn 70 g, Xylanase 50 g, phytase 750 FTU.

**Table 2.** Formulated nutrient composition (g/kg) and calculated digestible energy content (MJ/kg) of experimental diets (fresh basis).

Formulated composition (g/kg fresh basis)	**Control** ^a^	**Ideal 1** ^b^	**Ideal 2** ^c^	**Ideal 3** ^d^
**Crude protein**	175	150	150	150
**Oil A**	32.3	31.0	32.2	32.3
**Crude fiber**	38.5	32.5	35.6	35.7
**Ash**	52.1	45.7	45.5	45.6
**Lysine**	10.5	10.5	10.5	10.5
**Methionine**	3.3	3.6	3.4	3.4
**Methionine + Cysteine**	6.6	6.3	6.3	6.3
**Threonine**	6.7	7.0	7.0	7.0
**Tryptophan**	2.2	2.0	2.0	1.8
**Arginine**	10.8	8.4	8.3	8.3
**Isoleucine**	6.1	5.8	4.6	4.6
**Valine**	7.2	7.4	5.6	5.6
**Calcium**	8.8	8.5	8.5	8.5
**Phosphorus**	6.1	5.9	5.9	5.9
**Ca: P**	1.45	1.45	1.45	1.45
**Average phosphorus**	3.3	3.7	3.5	3.5
**Salt**	8	7	7	7
**Potassium**	6.4	5.2	5.1	5.1
**Digestible energy (MJ/d)**	13.85	13.85	13.85	13.85
**Amino acids ratios to lysine**				
**Methionine + Cysteine **	63	60	60	60
**Threonine**	63	67	67	67
**Tryptophan**	21	19	19	17
**Arginine**	103	80	79	79
**Isoleucine**	58	55	43	43
**Valine**	69	70	53	54

a Control: 175 g/kg crude protein (CP).

b Ideal 1: 150 g/kg CP with IP balanced to Lys, Met, Thr, Trp, Val, Ile and Arg.

c Ideal 2: 150 g/kg CP with IP balanced to Lys, Met, Thr and Trp.

d Ideal 3: 150 g/kg CP with IP balanced to Lys, Met, and Thr.

### Performance parameters and N excretion

To determine pig productivity, feed intake (FI) and average daily gain (ADG) were measured and feed conversion ratio (FCR) was calculated. Backfat depth at the P_2_ position (approximately 6.5 cm away from dorsal midline at the last rib curve (Tummaruk et al. 2009)) and cold carcass weight were recorded at a commercial abattoir. Kill-out percentage (KO %) was calculated (carcass weight/BW at slaughter × 100) to determine how much profitable carcass weight was obtained.

### Nitrogen retention was also calculated using the equation


(1) (ADG×0.16)/6.25

Where ADG × 0.16 is CP (Whittemore et al. 1988) and the conversion factor of 6.25 was used to convert CP to N retention. N excretion was then calculated by subtracting N retention values from N intake determined from formulated CP % of diets and feed intake (FI) at the pen level. FI was recorded every 3 wk on a pen basis. This was then used to calculate average FIs over 12 to 18 wk. The average daily feed intake calculated over 12 wk to slaughter, was used to calculate N intake.

### Fecal consistency scoring

A score of fecal consistency was conducted at the start (12 wk of age) and end (at slaughter) of the trial, which consisted of scoring the feces on the floor of each pen similarly to that described by Spitzer et al. (2014). The scoring system used was as follows:

1: Very soft/watery, 2: Soft, 3: Normal, 4: Hard, 5: Very hard.

Additionally, a score of “dirtiness” of the pigs’ skin was conducted every 3 wk at the time of weighing (the score at 12 wk was used as a baseline measure). Pig skin refers to the total amount of skin/flesh from all pigs in the pen. The scoring system was as follows:

1: Clean, 2: 25% dirty, 3: 50% dirty, 4: 75% dirty, 5: 100% dirty

### Economic evaluation

Economic return over feed costs for the experimental period were calculated for each dietary treatment using diet costs, ADG, FCR, kill-out yield (%) and income per kg of pig meat. The diet costs for control, Ideal diet 1, 2 and 3 were £250, £275, £252 and £241 per ton respectively. Income per kg of pig meat was taken as £1.55/kg, which was the typical price/kg in Northern Ireland at the time of writing.

### Statistical analysis

The data in this trial were analyzed by an analysis of variance using Genstat 8.0. The pen was considered the experimental unit and replicate was used as a blocking factor in the analysis. When testing the hypotheses, *P* values <0.05 were considered significant, and *P* values between 0.05 and <0.10 were considered to indicate a trend.

## Results

### Experimental diet analysis

Analyzed levels of nutrients, gross energy and AA in the experimental diets are shown in Table 3. There was good agreement between formulated and analyzed levels of CP, oil A, crude fiber, ash, and Lys, Met, Cys, Thr, Trp, and Arg. Formulated and analyzed levels of Ile and Val were extremely close for Ideal diet 1 (5.8 vs 6.1 g/kg for Ile and 7.4 vs 7.3 g/kg for Val), which was as expected as this diet was balanced down to these AAs. There was not such close agreement between the analyzed and formulated levels of Ile and Val for the other diets with analyzed levels being higher than formulated although still lower than the Control and Ideal 1 diet.

**Table 3.** Analyzed nutrient composition (g/kg) and energy content (MJ/kg) of the experimental diets (fresh basis).

Composition (g/kg)	**Control** ^a^	**Ideal 1** ^b^	**Ideal 2** ^c^	**Ideal 3** ^d^
**Dry matter**	887	884	885	887
**Crude protein**	179	158	150	144
**Crude fiber**	37	33	36	37
**Oil A**	32.2	31.9	32.5	33.0
**Oil B**	39.4	37.6	37.6	39.1
**Ash**	50.0	46	46	44.0
**Gross energy (MJ/kg)**	16.2	16.3	16.3	16.0
**Lysine**	10.4	10.6	10.5	10.6
**Methionine**	3.1	3.5	3.3	3.4
**Cysteine**	3.2	2.6	2.8	2.7
**Threonine**	6.5	6.7	6.5	6.5
**Tryptophan**	2.1	1.8	1.9	1.6
**Arginine**	10.9	8.9	8.4	8.2
**Isoleucine**	7.0	6.1	5.6	5.3
**Valine**	8.1	7.3	6.9	6.5
**Alanine**	7.9	6.7	6.6	6.4
**Aspartate**	15.8	12.8	11.7	11.2
**Glutamate**	33.3	28.8	27.6	27.2
**Glycine**	7.6	6.2	6.2	6.1
**Histidine**	4.4	3.7	3.5	3.5
**Leucine**	13.4	11.3	10.9	10.6
**Phenylalanine**	8.6	7.1	6.5	6.3
**Proline**	11.4	9.8	9.4	8.2
**Serine**	8.2	6.9	6.5	6.4
**Tyrosine**	4.4	3.0	3.5	2.7

a Control: 175 g/kg crude protein (CP).

b Ideal 1: 150 g/kg CP with IP balanced to Lys, Met, Thr, Trp, Val, Ile and Arg.

c Ideal 2: 150 g/kg CP with IP balanced to Lys, Met, Thr and Trp.

d Ideal 3: 150 g/kg CP with IP balanced to Lys, Met, and Thr.

### Production performance

The results of pig production performance are shown in Table 4. Reduced dietary CP levels had no significant effect on the feed intake (FI) of pigs at any stage (*P* > 0.05). However, ADG was increased in pigs in the 12 to 15 wk stage when offered low protein diets Ideal 1 and Ideal 2 compared to those offered the Control and Ideal 3 diets (*P* = 0.021). This is reflected in the body weight of pigs at 15 wk of age, as pigs offered diets Ideal 1 and 2 tended (*P* = 0.051) to be heavier than pigs offered the higher CP (control) diet. Diet had no significant effect on ADG or body weight at any other stages. Similarly, FCR was improved in pigs fed the Ideal 1 diet (2.08 g/g) compared to control pigs (2.41 g/g; *P* = 0.033) between weeks 12 and 15. However, these differences were not apparent at any other stage. Carcass cold weight was significantly higher in pigs fed the Ideal 1 diet (86.0 kg) compared to the other groups (Control: 82.7, Ideal 2: 84.0. and Ideal 3: 83.2 kg; *P* = 0.006). No other differences in carcass traits were observed.

**Table 4.** Growth performance, carcass traits and N balance of pigs offered the experimental diets.

Measure	Control^1^	Ideal 1^2^	Ideal 2^3^	Ideal 3^4^	S.E.M.	*P*-value
**Weight (kg)**						
** 12 wk**	39.5	40.3	40.5	39.9	0.43	0.394
** 15 wk**	53.8	56.8	56.4	55.5	0.74	0.051
** 18 wk**	72.6	75.2	75.1	72.1	1.11	0.140
** 21 wk**	93.3	96.6	95.9	93.6	1.36	0.242
** Slaughter^5^**	111.1	111.4	112.7	112.8	1.13	0.629
**Carcass traits**						
** Cold weight (kg)**	82.7^a^	86.0^b^	84.0^a^	83.2^a^	0.56	0.006
** Backfat (P_2_; mm)**	10.0	10.8	10.3	10.4	0.24	0.232
** Kill-out yield (%)**	74.9	76.6	75.8	75.0	0.46	0.087
**Feed intake**						
** 12 to 15 wk**	1634	1634	1661	1645	67.2	0.991
** 12 to 18 wk**	1836	1869	1847	1800	57.7	0.859
** 12 wk–slaughter**	2207	2227	2203	2275	62.0	0.838
**Average daily gain**						
** 12 to 15 wk**	682^a^	786^b^	755^b^	742^a,b^	21.4	0.021
** 12 to 18 wk**	786	830	823	766	22.3	0.168
** 12 wk–slaughter**	903	907	922	908	18.7	0.891
**Feed conversion ratio**						
** 12 to 15 wk**	2.41^b^	2.08^a^	2.20^a,b^	2.22^a,b^	0.071	0.033
** 12 to 18 wk**	2.34	2.25	2.25	2.37	0.053	0.273
** 12 wk to slaughter**	2.45	2.45	2.39	2.51	0.050	0.437
**N balance**						
** N Intake (g/d)**	61.8^b^	53.5^a^	52.9^a^	54.6^a^	1.57	0.003
** N Excretion (g/d)**	38.7^b^	30.2^a^	29.3^a^	31.4^a^	1.31	<.001
** N Retention (g/d)**	23.1	23.2	23.6	23.2	0.48	0.891

1 Control: 175 g/kg crude protein (CP).

2 Ideal 1: 150 g/kg CP with IP balanced to Lys, Met, Thr, Trp, Val, Ile and Arg.

3 Ideal 2: 150 g/kg CP with IP balanced to Lys, Met, Thr and Trp.

4 Ideal 3: 150 g/kg CP with IP balanced to Lys, Met, and Thr.

5 Slaughter weight (kg) of pigs at around 22 wk of age.

Means with different superscripts, “a and b” across rows are significantly different.

### Nitrogen intake, retention and excretion

A reduction in dietary CP in Ideal 1, 2, and 3 significantly reduced N intake (*P* = 0.003) of the pigs by 15% compared those offered the control diet (Table 4). N retention was not significantly affected by diet (*P* = 0.891), averaging 37.4% of N intake for the control diet, 43.4% of N intake for the lower CP diets. As a result of the reduced N intake and similar N retention, N excretion was significantly (*P* < 0.001) reduced from pigs offered the lower CP diets compared to those offered the control diet (average of 30.5 vs 40.1 g/d and an average of 24% reduction in N excretion).

### Fecal consistency and dirty pig scoring

**Fig. 1.**
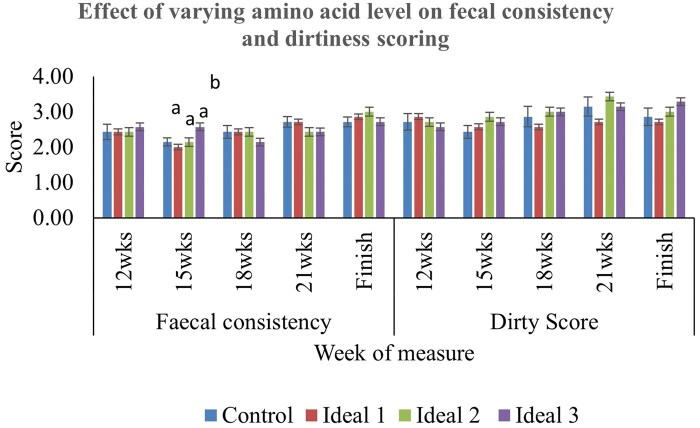
Effect of varying amino acid level on fecal consistency and dirtiness scoring of pigs. Error bars depict standard error of the mean. Treatment diets: Control: 175g/kg crude protein (CP), Ideal 1: 150g/kg CP with IP balanced to Lys, Met, Thr, Trp, Val, Ile and Arg, Ideal 2: 150g/kg CP with IP balanced to Lys, Met, Thr and Trp, Ideal 3: 150g/kg CP with IP balanced to Lys, Met, and Thr. ^a,b,c^Within each week, bars that do not share a common superscript are significantly different (*P* < 0.05).

Fecal consistency and dirty scoring are shown in Fig. 1. There was no dietary influence on dirtiness scoring throughout the study, and only fecal consistency at 15 wk differed between treatments; pigs fed the Ideal 3 diet had a higher consistency score (2.57) compared pigs from all other treatments (Control: 2.14, Ideal 1: 2.00, and Ideal 2:2.14; *P* = 0.016).

### Economic evaluation

Table 5 presents the return over feed for each dietary treatment for the experimental period. The lowest return over feed cost (£36.52) was for pigs offered the Ideal 1 diet and the highest return over feed was for pigs offered Ideal 2 and Ideal 3 diets (£41.35 and £40.65). The return over feed for pigs offered the control treatment was £39.26.

**Table 5.** Return over feed costs from pigs offered the experimental treatments (12 wk to slaughter).

	Control	Ideal 1	Ideal 2	Ideal 3
**Price per ton (£)**	250	275	252	241
**Pig days**	79.43	78.43	78.43	80.43
**FCR**	2.45	2.45	2.39	2.51
**p/kg gain (p)**	61.3	67.4	60.2	60.5
**Kg of gain (kg)**	71.6	71.1	72.2	72.9
**Cost of gain (£)**	43.86	47.90	43.48	44.10
**Kill-out yield (%)**	74.9	76.6	75.8	75.0
**Kg of pigmeat in experimental period (kg)**	53.6	54.5	54.7	54.7
**Price/kg gain dead weight (£)**	1.55	1.55	1.55	1.55
**Income/kg pigmeat in experimental period (£)**	83.12	84.42	84.83	84.75
**Return over feed/pig in experimental period (£)**	39.26	36.52	41.35	40.65

## Discussion

This study provides important information relative to diet modification in pigs to benefit the environment and economy, but maintain satisfactory pig growth and performance. Preliminary results indicated no difference in finishing pig performance among dietary treatments, which confirms that environmental benefits (24% reduction in N excretion) resulting from diet CP reduction can be achieved by precise amino acid supplementation. Not only that, but also nitrogen utilization efficiency was improved (high N retention percentage), meaning the amount of N excreted per kilogram of pork produced was reduced, highlighting the potential of low-CP diets to minimize environmental impacts while sustaining production efficiency. Pigs fed with IP levels balanced down to Ile and Val exhibited substantial gains in ADG increasing from 682 g/d in the control diet to 786 g/d in the Ideal 1 diet and FCR, improving from 2.41 in the control diet to 2.08 in the Ideal 1 diet during early finishing periods (12 to 15 wk), which demonstrates the crucial importance of these AA during early development stages. However, diets balanced down to Ile and Val showed no lasting performance benefits in the finishing stage, which may be due to an oversupply or adequate meeting of AA requirements at these stages. This suggests a complex interaction between dietary protein levels and AA ratios and the specific nutritional needs of pigs at different growth phases. This fine-tuning of amino acid supplementation highlights the need for strategic diet formulation in pig nutrition in order to improve performance without compromising environmental and economic sustainability. Feed cost-effectiveness analysis showed that the reduced CP diets without Ile and Val supplementation were the most cost-effective feed types, despite an amino acid supplementation that allowed a better early growth performance, but did not always justify these extra expenses. Indeed, this study revealed the need for balance between adequate nutrition and environmental and economic sustainability in meeting the industry’s goal of reduced environmental impact without sacrificing profitability and productivity.

When Ile and Val were allowed to “float” in the formulation, they were slightly higher than formulated in the Ideal diets 2 and 3 (ie those diets, which formulated to be lower than IP requirements for Ile and Val) though crucially they were still lower than the Control and Ideal diet 1 treatments (ie those diets, which were formulated to be at IP requirements for Ile and Val). There appeared to be a benefit of offering the reduced CP diet with IP formulated to Ile and Val in the early finisher stage in terms of ADG and FCR, which were not observed in the high CP, which was not balanced for Ile and Val. Evidence from Zhao et al. (2019) suggest that supplementation of Val (0.64 g/kg) is important in the earlier finisher stage in pigs (25 to 50 kg) and can improve FI and ADG. These authors placed Val as the next limiting AA after Trp for early finishing and Ile as the next limiting AA after Trp in the later finishing stage (50 kg+) as FI and ADG improved when diets were supplemented to contain IP levels of Val. The improvement in FCR and ADG in the early finishing stage with Ile and Val supplementation seen in the current study may be a result of the beneficial effect of these branched-chain AA on the production of glutamine. Glutamine has a function in plasma production, skeletal muscle development and immune cell energy supply (Self et al. 2004). Ile and Val also have a role to play in protecting intestinal morphology (Ren et al. 2015). While there was no apparent disease challenge to the pigs in the current study, the higher level of Ile and Val may have provided a boost to the immune system in the early finishing stage when pigs were randomized onto experimental treatment and mixed with unfamiliar penmates. However, despite some early benefits, there was no overall positive effect on performance over the entire finishing period as a result of formulating IP to Ile, and Val.

In the present study, we observed differences in amino acid ratios (ie Met + Cys: Lys and Thr: Lys ratio) between lower CP diets and the higher CP control diet. The lower Met + Cys: Lys ratio in lower CP diets was probably due to the lower amount of natural protein sources (soybean meal and rapeseed meal, which are rich in sulfur AAs) included in the lower CP diets. These were replaced by other feed ingredients (wheat and maize) that contain less sulfur AAs. In addition, although synthetic Met was added to meet minimum requirements, Cys was not added; hence, the Met + Cys: Lys ratio was lower. The higher Thr: Lys ratio in the lower CP diets was probably due to the additional Thr added to maintain the IP ratio. Thr is the third limiting amino acid in the pig diet, and its addition becomes even more important in lower CP diets due to the lower amount of natural protein sources. In the current diets, Thr was added in the lower CP diets to meet the protein synthesis for growth but it was not added in the higher CP diets. These results indicate the importance of AA supplementation in maintaining the IP ratio in lower CP diets.

Previous studies have investigated the effects of reducing CP content of the diet by 4 percentage units or more in gilt/castrate systems (Figueroa et al. 2003; Kerr et al. 2003). This study examined the response of pigs offered diets reduced in CP by 2.5 percentage units (25 g/kg) and while this may appear as a smaller reduction in dietary CP, the CP content of the low protein diet (150 g/kg) is similar to the concentrations in low protein diets of other studies (Powell et al. 2011; Gloaguen et al. 2014) and low for 40 kg pigs in gilt/entire male production systems. Indeed, offering the control diet (higher CP) beyond requirements represents an energy cost to the pig due to the need to deaminate the excess protein and excrete the excess nitrogen. Throughout the overall finishing period, there was no difference in performance as a result of offering lower CP diets with FI, ADG, FCR, and slaughter weight being more or less identical across treatments. Diets low in CP have been shown to cause increased backfat due to a number of reasons but the main reason being an imbalance between protein and energy meaning that fat is deposited (Wang et al. 2018), but this was not observed in this study as the backfat assessed by P_2_ was low for all pigs despite the difference in energy: protein between the control and lower CP diets. It is well established that if energy is over-supplied in relation to protein, an increase in fat deposition will occur and if protein is over-supplied in relation to energy, energy is required to deaminate the protein, which reduces lean deposition and essentially ADG (Patience et al. 2015).

A higher fecal consistency was reported in pigs offered the Ideal 3 diet at 15 wk compared to all other treatments, however there was no difference at any other stage. There is evidence to suggest that a reduction in CP content of feed can reduce the occurrence of post weaning diarrhea in young pigs through improvement in gut health (Heo et al. 2008; Zheng et al. 2016). Though the pigs used in this study had passed the nursery stage and their guts may already be matured enough to cope with high levels dietary CP. It could be for this reason why we see no overall effects of CP on fecal consistency in this study. It was hypothesized that the lower dietary CP could cause a reduction in water intake as there would be less excess CP to deaminate and excrete (Roux et al. 2011), which would in turn increase the dry matter of the feces and result in cleaner pigs. This was not observed in this study but the effect on water intake is not known as water usage was not measured.

This study provides evidence in support of those of Canh et al. (1998) and Hayes et al. (2004) and Ball et al. (2013) in that a reduction in dietary CP by 25 g/kg reduced both N intake and N excretion, without affecting N retention (or growth). Although the levels of N excretion in the current study are higher than those reported by Ball et al. (2013) due to heavier pigs, it does highlight that a reduction in dietary CP can significantly reduce the environmental impacts of pig production. N excretion was reduced by 24% through the 25 g/kg reduction in dietary CP, which is of significant environmental benefit and in keeping with the magnitude of N excretion reductions reported by others (Wang et al. 2018) when performance is maintained. Nitrogen retention was not different between treatments, indicating that the nitrogen supplied was adequate for nonessential amino acid (NEAA) synthesis. However, levels of some NEAAs (eg glutamate) were lower in the Ideal diets than in the control, which may be of concern for late performance or gut health. Future work should evaluate levels of NEAA in the low-CP diets to ascertain if NEAAs are adequate for protein synthesis and other physiological functions.

When an economic evaluation was conducted, the highest return over feed costs was from pigs offered the Ideal diets 2 and 3 as a result of lower diet costs in relation to the control. Ideal diet 1 was the most expensive due to the Ile and Val inclusion and also showed the lowest return over feed for pigs offered this diet, £2.74 lower than for pigs offered the control diet. This was due to a reduced kg per gain and increased kill-out percentage. The improved kill-out percentage is unlikely to be attributable to dietary CP level as the effect was not observed in pigs offered the other lower CP diets, which is in keeping with what has been reported by other workers Kerr et al. (1995) and Zhou et al. (2015). In order to maintain the balance in energy and CP and achieve IP down to Ile and Val, Ideal diet 1 contained lower levels of pollard and rapeseed meal (Table 1), which reduced the fiber (Table 3). Reduced fiber increases kill-out percentage as it reduces gut fill and gut weight, while increased fiber reduces the kill-out percentage by increasing the gut fill and gut weight (Clarke et al. 2018), this contributed to Ideal diet 1 pigs having a high carcasse weight (Table 4). Apart from the beneficial effect of dietary fiber on kill-out percentage the carcass weight advantage of Ideal 1 pigs was due to the balanced amino acid profile of the diet containing valine and isoleucine supporting lean tissue deposition during the early finishing period. This is reflected by the higher ADG and better FCR between 12 and 15 wk, as well as the lower nitrogen excretion showing efficient utilization of dietary protein for growth.

Another important finding is the substantial reduction in soyabean meal usage in the lower CP diets. For the optimum diets (Ideal diets 2 and 3) in terms of return over feed costs, the soyabean meal inclusion was reduced from 190 to 102 g/kg. If these diets were employed across the industry, this reduction would equate to 46% less soybean meal used for finishing pig diets; thus decreasing the demand for soybean. Soybean cultivation is linked to negative environmental impacts such as deforestation, land use changes and greenhouse gas emissions (Pendrill et al. 2019; Escobar et al. 2020). Although soybean, have the ability to fix atmospheric nitrogen, and hence require minimal synthetic N fertilizers (Jensen et al. 2020), their cultivation is resource intensive requiring phosphorous fertilizers and use of pesticides among other inputs, which contribute to green house gas emissions (Fearnside 2005; Searchinger et al. 2018). A reduced demand for soybean would mitigate the negative environmental impacts in addition to the N excretion reductions as a result of lower dietary CP.

This study has several limitations that could influence the results, and their generelazability to a broader context. First, we did not conduct a separate analysis for boars and gilts, which were housed in mixed-sex pens, as a result, potential differences in dietary responses could be hidden, especially, considering that boars have higher protein deposition needs, which could have affected ADG and FCR outcomes. Second, we did not evaluate whether nonessential AAs in low-CP diets were sufficient despite measuring their levels in the diet (eg glutamate: 27.2 to 28.8 g/kg vs 33.3 g/kg in control); the nonessential AA could have impacted late-stage performance and influenced fecal consistency due to potential deficiencies that restricted protein synthesis and gut function. Third, in the economic analysis, we did not evaluate the cost savings from reduced nitrogen (N) excretion. In the process we could have underestimated the absolute profitability of all low-CP diets (Ideal 1 to 3). Fourth, fluctuating ingredient levels that were adopted in this study could have introduced variability in amino acid availability due to differences in ingredient composition, thus potentially affecting the precision of amino acid balance and the interpretation of results. Despite these limitations, our findings offer useful guidance for developing affordable, low-CP diets that reduce environmental impact while sustaining performance levels in finishing pigs.

## Conclusions

This study provides evidence that the CP content of diets for finishing gilts and entire boars from 40 kg upwards can be reduced to 150 g/kg when IP is balanced to lysine, methionine, and threonine, maintaining production performance, reducing nitrogen (N) excretion by 24%, and significantly lowering soybean meal inclusion. Early finishing benefits were achieved by balancing IP to valine and isoleucine, but no overall advantage was observed at the end of the study. Despite the study limitations already highlighted, these findings demonstrate that even we can formulate diets that are both financially efficient and environmentally sustainable. Future research should address these limitations by conducting sex-specific analyses to optimize diets for boars and gilts, evaluating nonessential AA sufficiency to enhance late-stage performance and gut health, quantifying N excretion cost savings for accurate economic assessments, and considering formulating diets with a fixed base and supplementing synthetic AAs to achieve IP balance, thereby minimizing variability.

## Data Availability

Data for this study is available upon reasonable request from the corresponding author.
